# Bilateral Embryonic Vascular Persistence With Focal Aneurysm: Unveiling a Persistent Sciatic Artery Through Imaging: A Case Report

**DOI:** 10.7759/cureus.101843

**Published:** 2026-01-19

**Authors:** Krishna Vardhan, Gayathri M Sivagurunathan

**Affiliations:** 1 Radiology, Indira Gandhi Medical College & Research Institute, Puducherry, IND

**Keywords:** aneurysm, congenital vascular anomaly, gluteal swelling, persistent sciatic artery, vascular anomaly

## Abstract

Persistent sciatic artery is an uncommon embryologic remnant that typically remains clinically silent, yet has the potential to present dramatically when complicated. We report an unusual case of an 80-year-old woman who developed a progressively enlarging gluteal swelling over a year, associated with worsening pain in recent months. Further testing was necessary after ultrasonography revealed internal arterial flow within a cystic mass, despite the initial clinical impression of a soft-tissue neoplasm based on the clinical features. Computed tomography angiography (CTA) confirmed a persistent sciatic artery with a large, thrombosed aneurysm. An unexpected additional finding was the presence of a contralateral persistent sciatic artery, incompletely regressed and asymptomatic. Persistent sciatic artery pathology poses diagnostic difficulties. This case is one of the less common manifestations of this anomaly due to the combination of advanced age, bilateral arterial persistence, and a massive aneurysm. This report, hence, emphasizes the importance of keeping a wide differential diagnosis for gluteal masses and underscores the need for careful imaging evaluation to detect this rare but potentially serious vascular anomaly.

## Introduction

Persistent sciatic artery (PSA) is an exceptionally rare congenital vascular anomaly resulting from the failure of normal regression of the embryonic sciatic artery, a branch of the umbilical artery. During normal embryologic development, the sciatic artery, a dorsal branch of the umbilical-iliac system, supplies the developing lower limb. With proper growth of the superficial femoral artery (SFA) system, the sciatic artery typically involutes and disappears. In approximately 0.03% to 0.06% of the population, however, this regression does not occur, resulting in a persistent sciatic artery [1]. Bilateral PSA occurs in about 30% of cases [1]. Among symptomatic patients, aneurysm formation is the most common complication, reported in nearly 48% of cases [1]. The clinical importance of PSA stems from its predisposition to significant vascular complications. Atherosclerotic degeneration is more common in PSA than in normally developed arteries, making aneurysm formation the most frequent and well-documented complication [2]. Progressive enlargement, distal or proximal thromboembolism, and compression of nearby neural structures have all been reported as major complications [3]. In addition, because of its relatively superficial course within the gluteal region, the PSA is susceptible to repeated microtrauma from everyday activities such as prolonged sitting or inadvertent external pressure, potentially accelerating aneurysm development [3]. The objective of this report is to present a rare case of a PSA aneurysm presenting as a progressively enlarging gluteal mass in an elderly patient, highlighting the importance of imaging in accurate diagnosis and emphasizing the need to consider this rare vascular anomaly as a differential diagnosis for quicker diagnosis and accurate treatment before its progression into a complicated stage. 

## Case presentation

An 80-year-old woman presented with a one-year history of a progressively enlarging swelling in the right gluteal region associated with gradually worsening non-radiating pain over three months, with no characteristic symptoms suggestive of sciatica. She was hemodynamically stable at presentation with no known comorbidities. 

Local examination revealed a firm, tender mass with feeble pulsations in the right posterior gluteal region extending into the upper posterior thigh. The overlying skin was normal without warmth or discoloration. The right femoral pulse was diminished, although distal pulses in the right lower limb were palpable. Pulses in the left lower limb were normal. No signs of limb ischemia, edema, or neurological deficits were observed.

**Figure 1:**
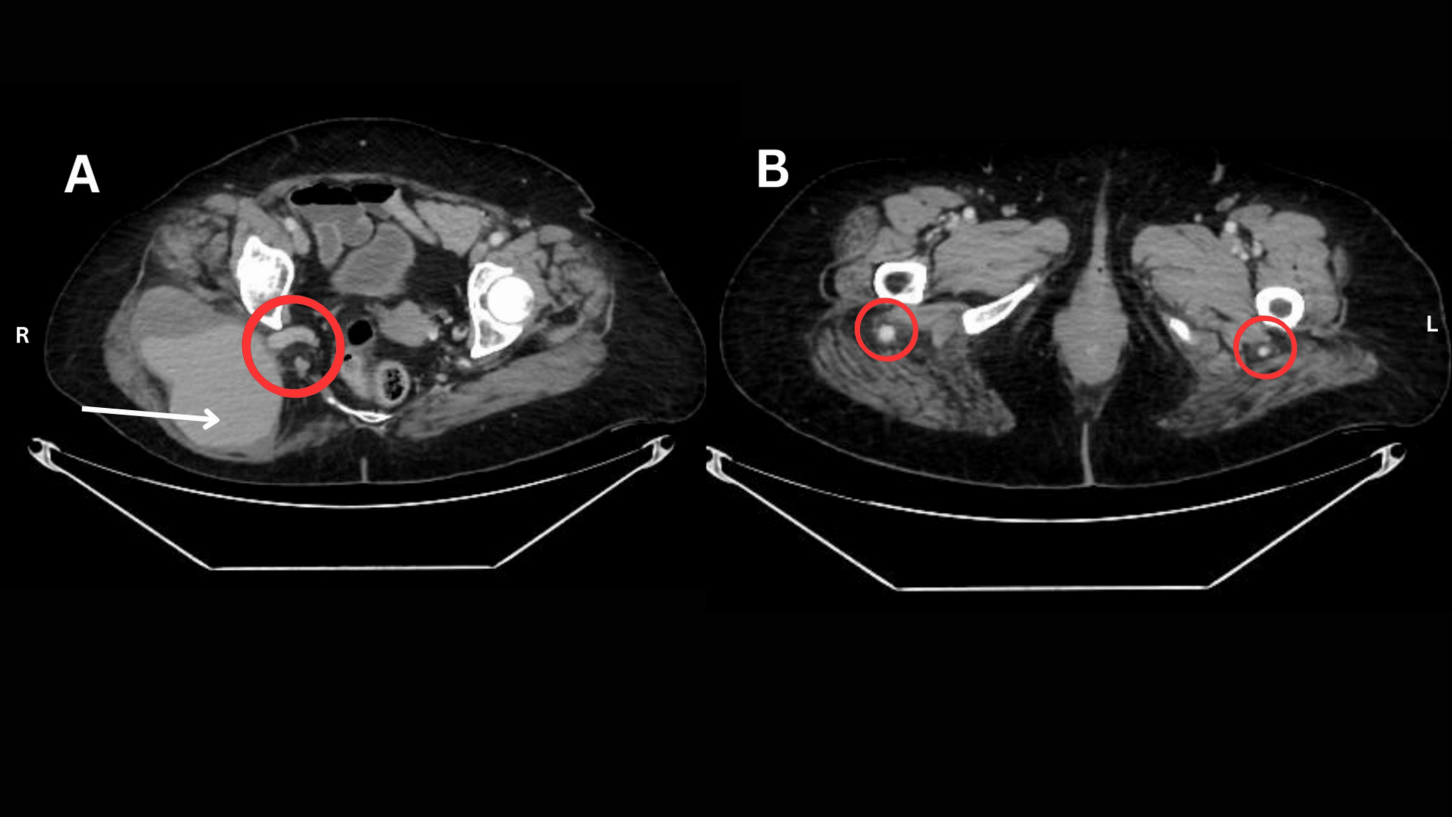
A) Axial CT angiogram at the upper gluteal region image: red circled region depicts the origin of the right PSA from the right internal iliac artery. The white arrow indicates a large PSA aneurysm measuring 11cm X 7cm with partial thrombosis. B) Axial CT angiogram at the lower gluteal region showing bilateral PSAs, with the left PSA displaying a relatively reduced luminal diameter compared with the right.

**Figure 2:**
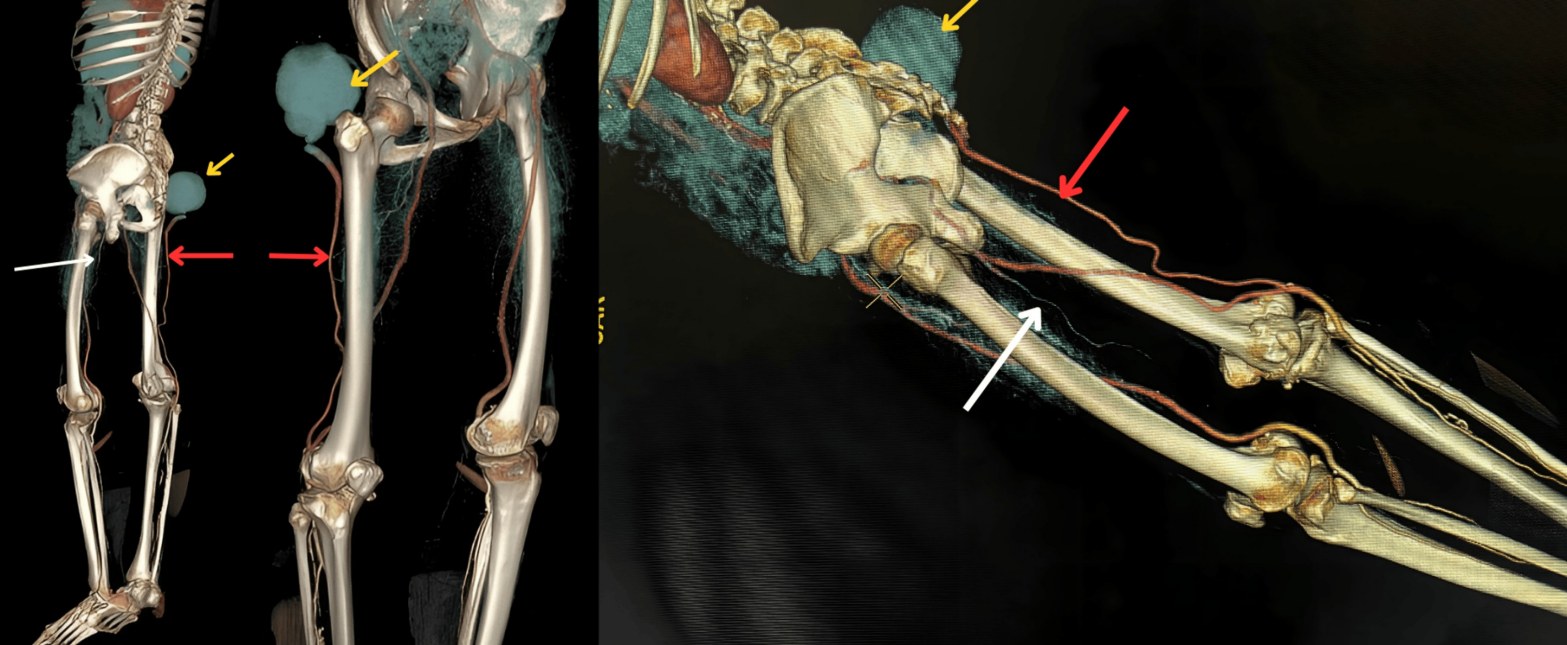
3D volume-rendered reconstruction image in which red arrows indicate the persistent sciatic arteries, white arrows denote the hypoplastic left PSA, and yellow arrows highlight the PSA aneurysm with mural thrombus.

**Figure 3:**
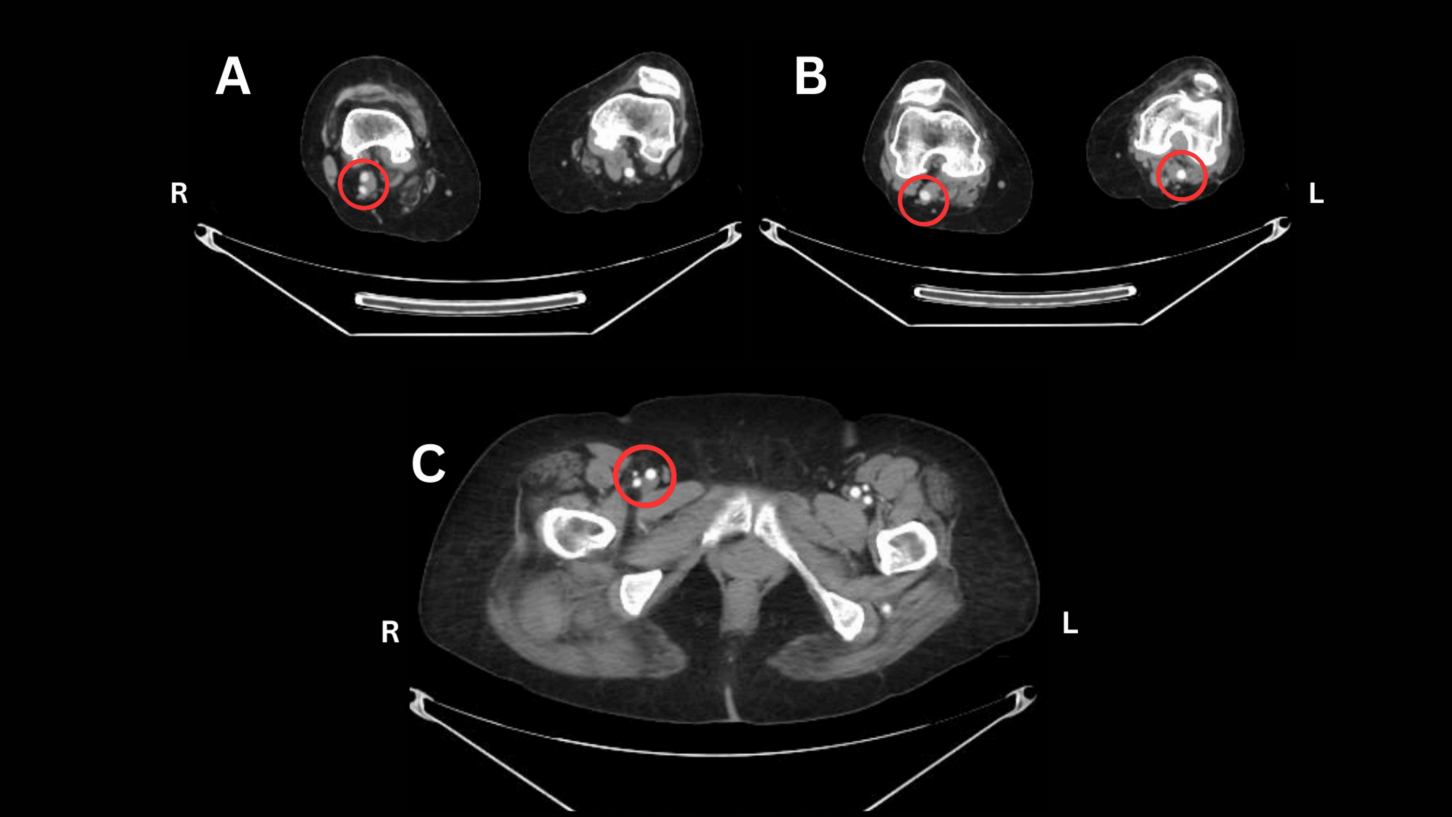
Axial CT angiogram image at the popliteal fossa level (A/B) Circled region illustrates the convergence of the superficial femoral artery (SFA) and the persistent sciatic artery (PSA) to form the popliteal artery (B). C) Axial CT angiogram at the inguinal region, shows the normal trifurcation of the common femoral artery (CFA) bilaterally, no anatomical variants are noted at the femoral level.

Given the interval increase in size, a soft-tissue sarcoma was initially suspected. Ultrasound of the swelling demonstrated a cystic lesion with internal arterial flow, suggesting an aneurysm. Contrast-enhanced CTA confirmed the presence of a persistent sciatic artery (PSA) with a large aneurysm measuring 11 cm × 7 cm containing intramural thrombus (Figure 1), and incidentally, the contralateral left PSA was noted descending to the distal 1/3rd of the hip, beyond which it is not visualized as annotated in image C of Figure 3. It is at the inguinal region, and the rest of the images are at the popliteal fossa. On the right side, both PSA and SFA were present, converging at the level of the popliteal fossa to form a single popliteal artery. Distal to this normal, cruel arterial branching, opacification of the crural ends was observed. The aneurysmal morphology and thrombotic burden are typical imaging features associated with long-segment PSA degeneration. Figure 2 represents 3D multiplanar CTA images that illustrate the course of the bilateral anomalous vascular structures from multiple viewing angles, allowing clear delineation of their course and anatomical relationships.

**Figure 4:**
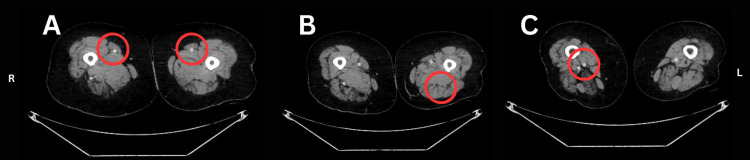
A) Axial CT angiogram at the proximal–mid one-third junction of the thigh, demonstrating superficial femoral arteries (SFA) of normal caliber without luminal narrowing. B) Axial CT angiogram at the adductor canal level, showing a left hypoplastic PSA with significantly reduced luminal diameter relative to the right. C) Axial CT angiogram at the distal one-third of the thigh, just below the adductor canal, where the circled region demonstrates normal caliber of the right SFA and right PSA at the popliteal level.

On the right side, both the PSA and the superficial femoral artery (SFA) are noted (Figure 3A), seen extending to the popliteal fossa and joining to form a single popliteal artery (Figure 3B), which likely accounted for the absence of ischemic symptoms despite the large aneurysm. Crural vessels (Figure 3C) were visualized up to the mid-third of the leg; beyond this, the anterior tibial artery was not seen, while the posterior tibial and fibular arteries were visualized up to the ankle. The right common femoral artery (CFA), SFA, and popliteal artery were of normal caliber (Figure 4C).

On the left side, the CFA and SFA were normal. A left-sided PSA was identified up to the distal third of the thigh, gradually tapering and becoming non-visualized distally (Figures 4A, 4B). The left SFA continued as the popliteal artery. Crural vessels were visualized up to the distal third of the leg, beyond which they were poorly delineated. 

According to the Pillet classification of persistent sciatic artery, as modified by Gauffre, the right-sided vascular anatomy corresponds to a Type III persistent sciatic artery, characterized by the existence of a persistent sciatic artery in which the proximal tract of the PSA persists with the normal femoral system. The left-sided anatomy is consistent with a Type I persistent sciatic artery, in which a complete sciatic artery is present alongside a normal femoral arterial system [4]. 

**Figure 5:**
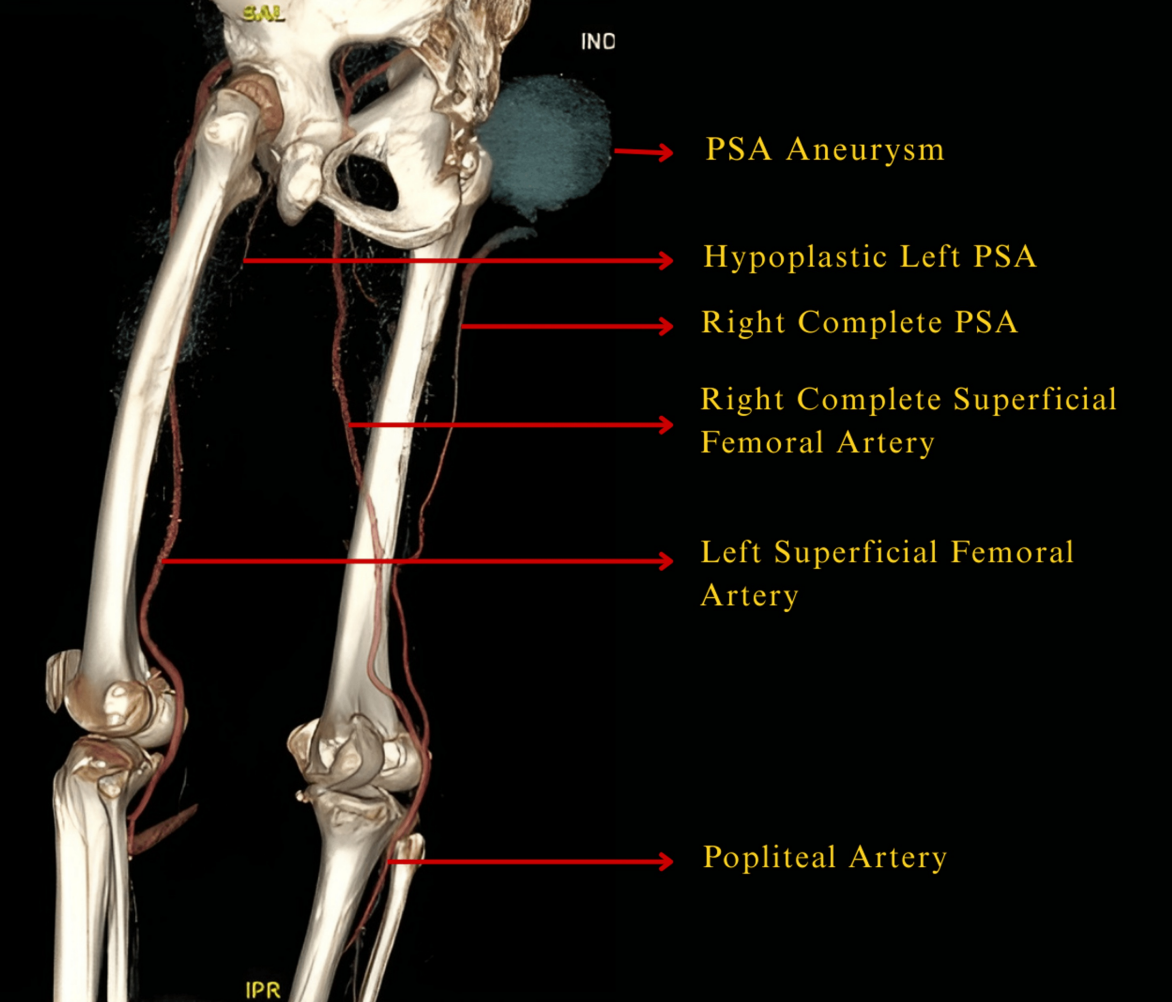
3D reconstructed image depicting the overall arterial anatomy, including the course of the bilateral PSAs.

Given the aneurysm’s size, associated symptoms, and risk of complications, the patient was referred to a tertiary center with endovascular expertise for further management. A labeled 3D reconstructed image (Figure 5) illustrates the bilateral persistent sciatic arterial anatomy, providing a concise overview of the vascular anomaly in this patient.

## Discussion

chPSA, a rare congenital vascular anomaly with aneurysmal degeneration, poses both diagnostic and therapeutic challenges [1]. During early embryonic development, the sciatic artery, arising from the umbilical artery, is the main blood supply to the lower limb. As the development progresses, the common and superficial femoral arteries form and replace the sciatic artery by the third month. Remnants of the sciatic artery also contribute to the gluteal, popliteal, and perineal arteries [1]. Many cases remain asymptomatic until complications arise, most commonly manifesting as a gluteal mass that may be mistaken for a soft-tissue neoplasm. Similar diagnostic confusion has been described in earlier reports and also occurred in this patient [4].

PSA has been traditionally classified based on its development and its anatomical relation with the SFA as type 1-5 by Pillet and Gauffre [5]. In 2016, Ahn et al. proposed a new system of classification incorporating symptomatology, the completeness of the PSA and SFA systems, and the presence or absence of aneurysmal change [6].

The clinical presentation in our patient, progressive right gluteal swelling with recent worsening of pain, is typical for aneurysmal degeneration of a PSA. The year-long history of a gradual enlargement followed by increasing pain suggests expansion of the aneurysm with possible wall stress, compression of the adjacent nerves, and impending rupture. The absence of lower-limb ischemia in our patient is explained by the arterial configuration, wherein both the PSA and SFA contributed to the formation of the popliteal artery. On the symptomatic side, this pattern corresponded to the ScPc morphology (complete SFA with incomplete PSA), classified as class Ia according to the Ahn-Min classification of persistent sciatic arteries [6]. By contrast, complete PSA variants commonly coexist with an underdeveloped SFA and therefore carry a much higher risk of limb-threatening ischemia [7].

The uniqueness of this case lies in the presence of bilateral PSA in an elderly patient with a large aneurysm on the right side and incompletely regressed, asymptomatic PSA on the left side. Although PSA aneurysms have been reported across a wide age range, presentation in elderly individuals is uncommon. Furthermore, aneurysms exceeding 10 cm are rarely documented. Only isolated reports describe PSA aneurysms of comparable size, including a 12-cm PSA aneurysm reported by Knight BC and Tait WF, endovascular management of PSA aneurysms in octogenarian patients documented by Koyama et al., and the report of an aneurysmal PSA in an 89-year-old woman described by Ooka T et al. [8-10].

This makes our case, an 80-year-old woman with an 11 cm aneurysm, an important addition to the limited literature addressing extremes of age, bilateral involvement, and aneurysm size in PSA pathology.

The main limitation of this case report is the lack of clinical follow-up and treatment outcome details, which hinders assessment of prognosis and management effectiveness. 

Imaging plays a central role in the diagnosis and management of PSA and its complications. Ultrasonography is useful for initial screening, demonstrating the vascular nature of the gluteal mass and providing a preliminary assessment of flow characteristics. Cross-sectional imaging with CTA and magnetic resonance angiography (MRA) can provide anatomical details, including the aneurysm’s size, extent, relationship to adjacent structures, and associated vascular supply patterns [2]. Conventional digital subtraction angiography (DSA) remains the gold standard owing to its superior depiction of distal arterial anatomy, feeding vessels, and aneurysm morphology, information that is critical for therapeutic planning.

With improvements in endovascular technology, the management of PSA aneurysms has changed significantly. Treatment depends on factors such as the size and shape of the aneurysm, the PSA type, the presence of limb ischemia, and the patient’s overall condition. Endovascular repair is now often preferred because it is less invasive and offers good results in suitable patients. However, open surgery is still needed when the anatomy is complex, the aneurysm is very large or infected, or endovascular access is difficult. In some cases, a combined (hybrid) approach may be used. Overall, the treatment plan should be tailored to each patient to prevent complications like rupture or embolization.

## Conclusions

PSA aneurysms, though rare, pose significant diagnostic difficulty and risk for serious complications. Clinicians should maintain a high index of suspicion when evaluating gluteal swellings. Early imaging and accurate diagnosis are essential to prevent limb-threatening or life-threatening sequelae.

What makes this case unique is the incidental detection of a contralateral persistent sciatic artery in an elderly patient, coupled with an exceptionally large aneurysm, highlighting the need for tailored diagnostic and therapeutic strategies in elderly individuals with atypical presentations. Longitudinal studies assessing the progression of asymptomatic PSA, especially contralateral or incidental findings as in our case, would provide valuable insight into surveillance strategies and risk factors for aneurysm formation. 
